# The Feasibility and Reliability of Upper Arm–Worn Apple Watch Heart Rate Monitoring for Surgeons During Surgery: Observational Study

**DOI:** 10.2196/50891

**Published:** 2023-11-01

**Authors:** Kazunosuke Yamada, Yasuaki Enokida, Ryuji Kato, Jun Imaizumi, Takahiro Takada, Hitoshi Ojima

**Affiliations:** 1 Department of Gastroenterological Surgery Gunma Prefectural Cancer Center Oota City, Gunma Japan

**Keywords:** Apple Watch, heart rate, surgery, robot

## Abstract

**Background:**

Health care professionals, particularly those in surgical settings, face high stress levels, impacting their well-being. Traditional monitoring methods, like using Holter electrocardiogram monitors, are impractical in the operating room, limiting the assessment of physicians’ health. Wrist-worn heart rate monitors, like the Apple Watch, offer promise but are restricted in surgeries due to sterility issues.

**Objective:**

This study aims to assess the feasibility and accuracy of using an upper arm–worn Apple Watch for heart rate monitoring during robotic-assisted surgeries, comparing its performance with that of a wrist-worn device to establish a reliable alternative monitoring site.

**Methods:**

This study used 2 identical Apple Watch Series 8 devices to monitor the heart rate of surgeons during robotic-assisted surgery. Heart rate data were collected from the wrist-worn and the upper arm–worn devices. Statistical analyses included calculating the mean difference and SD of difference between the 2 devices, constructing Bland-Altman plots, assessing accuracy based on mean absolute error and mean absolute percentage error, and calculating the intraclass correlation coefficient.

**Results:**

The mean absolute errors for the whole group and for participants A, B, C, and D were 3.63, 3.58, 2.70, 3.93, and 4.28, respectively, and the mean absolute percentage errors were 3.58%, 3.34%, 2.42%, 4.58%, and 4.00%, respectively. Bland-Altman plots and scatter plots showed no systematic error when comparing the heart rate measurements obtained from the upper arm–worn and the wrist-worn Apple Watches. The intraclass correlation coefficients for participants A, B, C, and D were 0.559, 0.651, 0.508, and 0.563, respectively, with a significance level of *P*<.001, indicating moderate reliability.

**Conclusions:**

The findings of this study suggest that the upper arm is a viable alternative site for monitoring heart rate during surgery using an Apple Watch. The agreement and reliability between the measurements obtained from the upper arm–worn and the wrist-worn devices were good, with no systematic error and a high level of accuracy. These findings have important implications for improving data collection and management of the physical and mental demands of operating room staff during surgery, where wearing a watch on the wrist may not be feasible.

## Introduction

Health care professionals have a significantly higher risk of burnout and work-life dissatisfaction compared to other professionals, as has been widely reported internationally [1-3]. Unfortunately, in the medical field, the primary focus has been on the well-being of patients, while the well-being of medical personnel has been largely ignored [4]. Operating room staff in particular are known to be highly stressed professionals, both physically and mentally, but have rarely been properly assessed due to the difficulty of wearing Holter electrocardiogram monitors or blood pressure monitors during surgery. In recent years, wrist-worn devices capable of monitoring heart rate (HR) have undergone remarkable development, allowing data to be collected in a variety of environments. These devices are useful for detecting atrial fibrillation [5], and health care professionals are often seen wearing them to monitor their HR during work, except during certain procedures where hygiene and sterility must be maintained. The Apple Watch (Apple) is the most reliable device, with the highest validity (ie, the smallest margin of error) of all smartwatches capable of measuring HR with Food and Drug Association Class 2 medical device certification [6,7]. The Apple Watch uses photoplethysmography technology to measure HR. This method involves shining green LED lights onto the skin and detecting the amount of light that is absorbed and reflected by the blood vessels in the wrist. As the heart beats, blood flow changes, causing a slight variation in the amount of reflected light. This variation is used to calculate the HR.

Meanwhile, the field of robotic-assisted surgery has also made remarkable progress, with its use steadily increasing in several areas of medicine. Meta-analyses have shown the safety and efficacy of robotic and laparoscopic approaches in patients undergoing curative surgery for rectal cancer [8]. The da Vinci surgical system consists of a surgeon’s console outside the sterile field and a patient-side cart within the sterile field. The surgeon uses the controls on the console to maneuver the robotic arm on the patient-side cart. Therefore, some robotic surgeons typically perform surgical maneuvers with a wristwatch on. On the other hand, wristwatches cannot be worn during laparoscopic surgery or laparotomy, where the surgeon needs to enter an aseptic field. However, if this device works when worn on the upper arm of the surgeon, where sterilization is not required, data collection can be expected to improve dramatically.

In this study, we examined the measurement error between 2 Apple Watches worn on the wrist and upper arm while the surgeon was using the console. This study aims to establish the correlation between upper arm and wrist HR measurements in the context of robotic-assisted surgery. The potential applicability of this correlation for general surgical scenarios, where upper arm monitoring could replace wrist monitoring, will improve data collection from operating room staff during surgery.

## Methods

### Device and Data Collection

**Figure 1:**
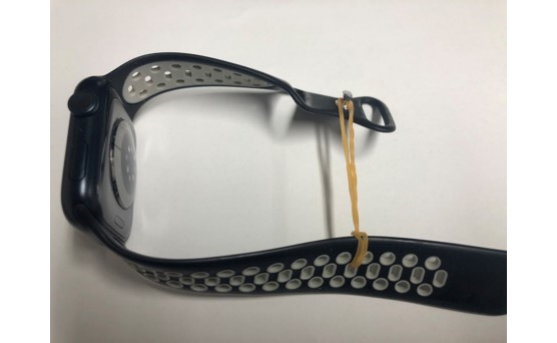
Adjustment of Apple Watch band length using a rubber band.

In this study, 2 identical Apple Watch Series 8 devices (45 mm) were used to monitor the HR of surgeons during robotic-assisted surgery. The surgeons performing the robotic-assisted surgery wore an Apple Watch on each wrist and upper arm and used the controls on the console to maneuver the robotic arm on the patient-side cart. When the Apple Watch was attached to the upper arm, the band was lengthened using rubber bands to adjust to a position causing the least discomfort to the surgeon (Figure 1).

**Figure 2:**
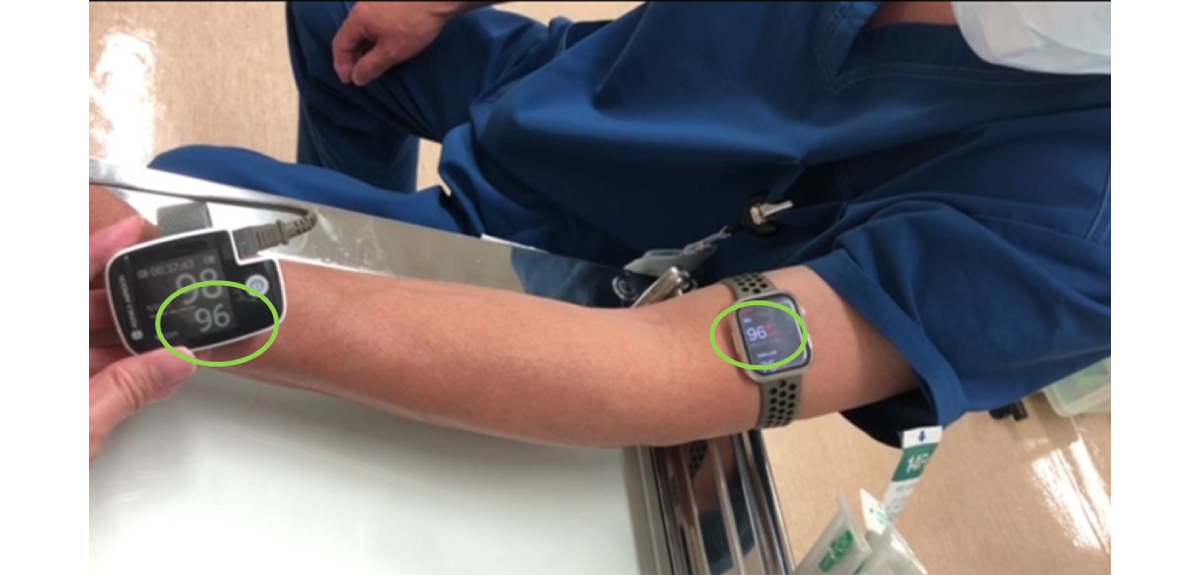
Positioning of the Apple Watch on the surgeon’s upper arm and comparison of heart rate readings with a fingertip pulse oximeter.

In this adjusted position, the watch was ideally located directly over the superficial vein of the upper arm. The readings were then compared to those from a fingertip pulse oximeter to ensure general consistency (Figure 2).

This study included monitoring the HR of surgeons during the first hour of console control. HR data were collected using the Hachi app provided by APTECH, which enabled the collection and extraction of HR data at 1-minute intervals from multiple Apple Watches on a single iPad (Apple). This app also facilitated centralized data management. The timing for initiating HR measurement within this 1-minute interval was not determined by the examiner’s discretion but was dependent on the app’s functionality. In addition to HR data, demographic information, such as gender, age, weight, height, and body mass index (BMI), was collected for all surgeons. Other information recorded included console time, operative time, and the type of surgical procedure performed. Wearing an Apple Watch on the upper arm is not a method recommended by Apple, and there are no reports evaluating the concordance of HR measurements between 2 Apple Watches simultaneously worn by the same individual at different anatomical sites. As an additional step to evaluate the results obtained in the study, HR data were also collected from a single surgeon by wearing both Apple Watches on both wrists using the same method. The purpose of this supplementary data collection was to check if the observed differences in HR measurements between the upper arm and wrist positions were within an acceptable range.

### Ethical Considerations

Informed consent was obtained from all the participants. This study was conducted in accordance with the ethical standards of the Helsinki Declaration of 1975. Ethics approval was obtained from the Ethics Committee of the Gunma Prefectural Cancer Center (405-04030).

### Statistical Analysis

The mean difference (MD) and SD of difference (SDD) between the wrist-worn and the upper arm–worn Apple Watches were calculated, and Bland-Altman plots were constructed to exclude systematic errors. Bias (MD) and limits of agreement (LoA; MD ± 1.96 × SDD) were plotted on the Bland-Altman plots to assess clinical applicability. The accuracy of the HR measurement from the Apple Watch worn on the upper arm was assessed based on the mean absolute error (MAE) and mean absolute percentage error (MAPE) between the upper arm and the wrist. MAE reflects the average size of the differences between predicted and observed values and ranges from zero to infinity, where lower MAE values indicate better forecasting performance. MAPE is commonly used as a measure of the prediction accuracy of a forecasting method. It is an average of the absolute values of the errors divided by the observed values. MAPE ranges from 0% to 100%, where lower MAPE values indicate better predictive performance of the model. In general, a MAPE of less than 10% is considered highly predictive [9].

The intraclass correlation coefficient (ICC) was calculated to determine the correlation between the Apple Watch on the upper arm and the one on the wrist. ICC estimates and their 95% CIs were calculated using SPSS statistical package version 22 (SPSS) based on a single rater (k=1), consistency, and a 2-way mixed-effects model.

Based on the 95% CI of the ICC estimate, values <0.5, between 0.5-0.75, between 0.75-0.9, and >0.90 were considered to be indicative of poor, moderate, good, and excellent reliability, respectively [9]. For all statistical tests, the alpha level adopted for significance (2-tailed) was set at *P*<.05.

## Results

The trial involved 4 surgeons with expertise in esophageal, gastric, and colorectal cancers. No surgeon had any medical or medication history, including arrhythmias. Additional characteristics of the surgeons are shown in Table 1.

All participants were informed by the investigator before surgery that the Apple Watch could be removed at the surgeon’s discretion after the 1-hour measurement, but they all continued to wear both Apple Watches until the console-based procedure was completed. The numerical test results are summarized in Table 2.

**Figure 3:**
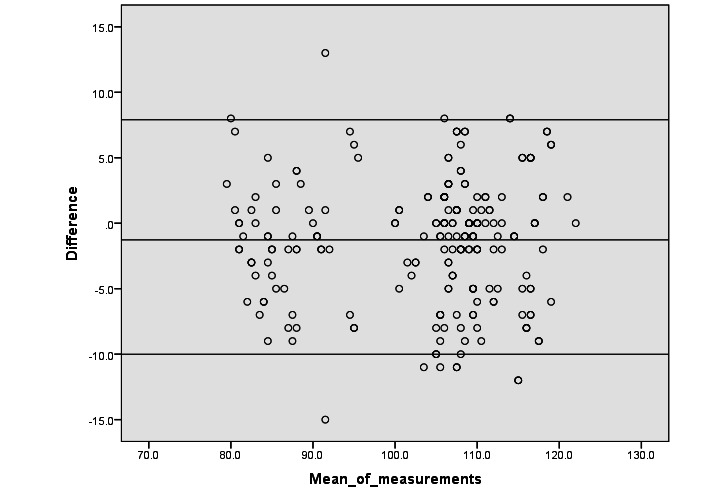
Evaluation of heart rate agreement between wrist-worn and upper arm–worn Apple Watches across the whole group using Bland-Altman plots. No significant systematic error was observed.

The SDDs for the whole group and participants A, B, C, and D were 4.66, 4.53, 3.66, 4.91, and 4.73, respectively, and the biases (lower and upper LoAs) were −1.275 (−10.01 and 7.90), −1.75 (−10.62 and 7.13), 0.933 (−8.1 and 6.24), −1.433 (−11.06 and 8.19), and −2.85 (−12.12 and 6.42), respectively. Bland-Altman plots and scatter plots showed no systematic error when comparing the HR measurements obtained from the upper arm–worn and wrist-worn Apple Watches (Figure 3).

The MAEs for the whole group and participants A, B, C, and D were 3.63, 3.58, 2.70, 3.93, and 4.28, respectively, and the MAPEs were 3.58%, 3.34%, 2.42%, 4.58%, and 4.00%, respectively. The ICCs for participants A, B, C, and D were 0.559, 0.651, 0.508, and 0.563, respectively (*P*<.001). Following the previously mentioned limits, this can be interpreted as having moderate reliability.

Supplementary data were collected from a single surgeon who wore Apple Watches on both wrists (instead of the upper arm) using the same method. The SDD was found to be 7.17, and the bias (lower and upper LoA) was 2.1 (−11.95 and 16.15). The MAE was 6.43, and the MAPE was 6.1%. The ICC was 0.025 *(P*=.42), which suggests poor agreement between the 2 measurements.

**Table 1:** Characteristics of the surgeons.

Characteristic	Participant A	Participant B	Participant C	Participant D
Age (years)	60	42	40	38
Gender	Male	Male	Male	Male
Body mass index	24.7	30.3	18.3	21.9
Wrist circumference (cm)	18	19	16	16
Upper arm circumference (cm)	26	33	23.5	24.5
Surgical specialty	Esophageal cancer	Gastric cancer	Colorectal cancer	Colorectal cancer
Experience with robotic surgery (years)	4	0.2	1	2

**Table 2:** Comparison of heart rate measurements between upper arm–worn and wrist-worn Apple Watches.

Measurement	All participants	Participant A	Participant B	Participant C	Participant D	2 wrist-worn devices^a^
MD^b^ (bpm)	–1.275	–1.75	0.933	­–1.433	–2.85	2.10
SDD^c^ (bpm)	4.66	4.53	3.66	4.91	4.73	7.17
Lower LoA^d^ (bpm)	–10.01	–10.62	­–8.1	–11.06	–12.12	–11.95
Upper LoA (bpm)	7.90	7.13	6.24	8.19	6.42	16.15
MAE^e^	3.63	3.583	2.70	3.93	4.28	6.43
MAPE^f^ (%)	3.58	3.34	2.42	4.58	4.00	6.10
ICC^g^ (*P* value)	0.96 (<.001)	0.559 (<.001)	0.651 (<.001)	0.508 (<.001)	0.563 (<.001)	0.025 (.42)
Difference in measurement time (seconds)	N/A^h^	23	28	10	6	26

^a^Supplementary data were collected from a single surgeon who wore Apple Watches on both wrists instead of the upper arm.

^b^MD: mean difference.

^c^SDD: standard deviation of difference.

^d^LoA: limits of agreement.

^e^MAE: mean absolute error.

^f^MAPE: mean absolute percentage error.

^g^ICC: intraclass correlation coefficient.

^h^N/A: not applicable.

## Discussion

### Principal Findings

The Bland-Altman plots, MAEs, MAPEs, and ICCs were the statistical measures used to evaluate the agreement and reliability of measurements in this research on wrist-worn devices capable of monitoring HR. The study found a small bias between the upper arm–worn and wrist-worn devices, no systematic error, and a high predictive value for MAPE and moderate predictive value for ICC for each participant. It was concluded that there is good agreement and reliability of the measurements obtained by the Apple Watch when comparing the upper arm–worn device with the wrist-worn device.

Unexpectedly, the least agreement between the 2 devices was found in the supplementary data involving a surgeon wearing an Apple Watch on each wrist in the correct manner. It was assumed that having the watches worn correctly on both wrists would provide the most accurate and reliable measurements. However, experiments where the watch was worn on upper arm and wrist on the same side showed that hand movements were generally consistent, enabling more stable measurements. In contrast, when the watch was worn on both wrists, the left and right hand movements were completely different, which could have resulted in a significant discrepancy in the measured values. In robotic surgery, where the robot’s arms can bend beyond the natural range of human wrist motion, surgeons often bend their wrists to the limit. We hypothesize that this extreme movement may reduce venous blood flow, thereby increasing the likelihood of discrepancies in HR measurements. It is important to note that this is a speculation, and further studies are needed to confirm the cause of this unexpected result. Nonetheless, this finding highlights the importance of understanding the limitations and potential sources of error when using wearable devices for health monitoring purposes.

HR is associated with survival in both healthy individuals and patients with various underlying cardiovascular diseases [10-12]. For example, a resting HR above 75 beats per minute in healthy individuals is known to increase the risk of sudden death from myocardial infarction [13]. Additionally, experiencing stressful life events increases the risk of developing cardiovascular disease [14].

An increase in HR leads to a decrease in diastolic time and an increase in systolic time, resulting in decreased myocardial perfusion and increased left ventricular work. These changes can ultimately lead to left ventricular hypertrophy, myocardial damage, and congestive heart failure. Increased HR may also be associated with endothelial damage, oxidative stress, inflammation, and vascular stiffness, which can contribute to aging, the development of atherosclerosis, arterial hypertension, and a stiff aorta. An increase in pacing rate from 60 to 90 beats per minute in humans has been shown to reduce the distensibility of the carotid and radial arteries [15]. Moreover, acute stress can cause sympathetic nervous system activation and parasympathetic nervous system suppression, leading to greater myocardial contraction and an increased HR. This can also cause an increased inflammatory (IL-6) response due to altered autonomic nervous system activity, which is associated with an increased risk of cardiovascular disease [16,17]. Given these associations, it is considered important to monitor the HR of health care professionals. However, the specific nature of their work makes this very difficult, and very little research has been done in this area.

In our experiment, we took care to position the Apple Watch directly above the blood vessels on the body surface when it was worn on the upper arm. We could confirm that there was never an instance in which HR measurement failed during the experiment when the watch was worn on either the upper arm or the wrist. While Apple Watch HR measurements have generally been found to be accurate, there are several factors known to cause significant errors in the readings. One of the most common factors is when the Apple Watch is not worn snugly on the wrist. Accuracy can also be significantly reduced during high-impact activities, such as running or cycling [18]. Additionally, the darker the skin tone, the less accurate the readings are. It has also been suggested that accuracy may be reduced in obese people due to increased subcutaneous fat thickness [18,19]. Contrasting previous literature that points toward higher BMI as a source of measurement errors, our study challenges this notion. Specifically, Participant A (BMI 24.7) and Participant B (BMI 30.3) yielded reliable HR measurements. In contrast, Participant C, with a lower BMI of 18, produced measurements that were somewhat less reliable when compared to the other participants. For instance, Participant C’s scant subcutaneous fat could have hindered the Apple Watch’s skin adherence, compromising the accuracy of measurements. This leads us to consider that both extremes of body composition—be it obesity or leanness—could challenge the reliability of wrist-worn HR monitors like the Apple Watch. Finally, the HR per minute was recorded simultaneously on 2 Apple watches, but the measurement times were dependent on the Apple Watch and could not be matched exactly. This resulted in a potential 28-second measurement error. This discrepancy in measurement times may be one of the reasons why the measurements did not match exactly.

The study suggests that the Apple Watch, worn on the upper arm, could be used to measure the HR of health care professionals in confined surgical environments without the need for disinfection. This would make mental and physical stress monitoring convenient and reliable. This study is the first to use an Apple Watch worn on the upper arm to measure the HR of surgeons during surgery. The findings suggest that wearable devices, such as the Apple Watch, could be used to measure the HR of health care workers during surgical procedures where there are limitations in measuring vital signs. This can enable an analysis of specific time periods and provide a more focused understanding of how HR is affected during this critical period of the surgical procedure. However, it is important to note that the feasibility of using the upper arm placement may be compromised in activities requiring extensive movement. In such scenarios, the device may become dislodged, thereby affecting the reliability of HR measurements. Consequently, we recommend reserving upper arm mounting for specific, controlled environments, such as surgeries that involve a limited range of motions, similar to those associated with surgical operations.

### Limitations

This study has some limitations that should be considered. First, the sample size was small, and the inclusion of only 4 male Japanese doctors in robotic surgery may have led to selection bias. Therefore, the findings may not be generalizable to a wider population. Second, the method of wearing the Apple Watch on the upper arm for HR measurement is not recommended by Apple and was only evaluated within the limited range of movements during surgical procedures. Therefore, it may not be suitable for other types of physical activities or movements. Third, the timing of the measurements could not be exactly matched between the 2 Apple Watches, making the data less consistent.

### Conclusion

Our study showed that the HR measurements obtained from an Apple Watch worn on the upper arm during robotic-assisted surgery were moderately correlated and consistent with the measurements obtained from an Apple Watch worn on the wrist. The MAE and MAPE between the 2 positions were low, indicating an acceptable level of correlation and a high level of accuracy. Our findings suggest that the upper arm is a viable alternative to the wrist for monitoring HR during surgery when it is not feasible to wear a watch on the wrist. These findings have important implications for improving data collection and management of the physical and mental demands of operating room staff during surgery, where wearing a watch on the wrist may not be feasible.

## Abbreviations

HR: heart rate
ICC: intraclass correlation coefficient
LoA: limits of agreement
MAE: mean absolute error
MAPE: mean absolute percentage error
SDD: standard deviation of difference

## Data Availability

We confirm that the data supporting the findings of this study are available within the article and its supplementary materials. Additionally, the raw data that were analyzed during this study are available from the corresponding author upon reasonable request, provided that any necessary ethical approval is obtained and in compliance with applicable laws and regulations.
